# *In silico* Design for Systems-Based Metabolic Engineering for the Bioconversion of Valuable Compounds From Industrial By-Products

**DOI:** 10.3389/fgene.2021.633073

**Published:** 2021-03-26

**Authors:** Albert Enrique Tafur Rangel, Wendy Ríos, Daisy Mejía, Carmen Ojeda, Ross Carlson, Jorge Mario Gómez Ramírez, Andrés Fernando González Barrios

**Affiliations:** ^1^Grupo de Diseño de Productos y Procesos, Department of Chemical and Food Engineering, Universidad de los Andes, Bogotá, Colombia; ^2^Grupo de Investigación CINBIOS, Department of Microbiology, Universidad Popular del Cesar, Valledupar, Colombia; ^3^Center for Biofilm Engineering, Montana State University, Bozeman, MT, United States

**Keywords:** systems metabolic engineering, transcriptomics, machine learning, adaptive laboratory evolution, metabolic modeling resources/frameworks

## Abstract

Selecting appropriate metabolic engineering targets to build efficient cell factories maximizing the bioconversion of industrial by-products to valuable compounds taking into account time restrictions is a significant challenge in industrial biotechnology. Microbial metabolism engineering following a rational design has been widely studied. However, it is a cost-, time-, and laborious-intensive process because of the cell network complexity; thus, it is important to use tools that allow predicting gene deletions. An *in silico* experiment was performed to model and understand the metabolic engineering effects on the cell factory considering a second complexity level by transcriptomics data integration. In this study, a systems-based metabolic engineering target prediction was used to increase glycerol bioconversion to succinic acid based on *Escherichia coli*. Transcriptomics analysis suggests insights on how to increase cell glycerol utilization to further design efficient cell factories. Three *E. coli* models were used: a core model, a second model based on the integration of transcriptomics data obtained from growth in an optimized culture media, and a third one obtained after integration of transcriptomics data from adaptive laboratory evolution (ALE) experiments. A total of 2,402 strains were obtained with fumarase and pyruvate dehydrogenase being frequently predicted for all the models, suggesting these reactions as essential to increase succinic acid production. Finally, based on using flux balance analysis (FBA) results for all the mutants predicted, a machine learning method was developed to predict new mutants as well as to propose optimal metabolic engineering targets and mutants based on the measurement of the importance of each knockout’s (feature’s) contribution. Glycerol has become an interesting carbon source for industrial processes due to biodiesel business growth since it has shown promising results in terms of biomass/substrate yields. The combination of transcriptome, systems metabolic modeling, and machine learning analyses revealed the versatility of computational models to predict key metabolic engineering targets in a less cost-, time-, and laborious-intensive process. These data provide a platform to improve the prediction of metabolic engineering targets to design efficient cell factories. Our results may also work as a guide and platform for the selection/engineering of microorganisms for the production of interesting chemical compounds.

## Introduction

**FIGURE 1:**
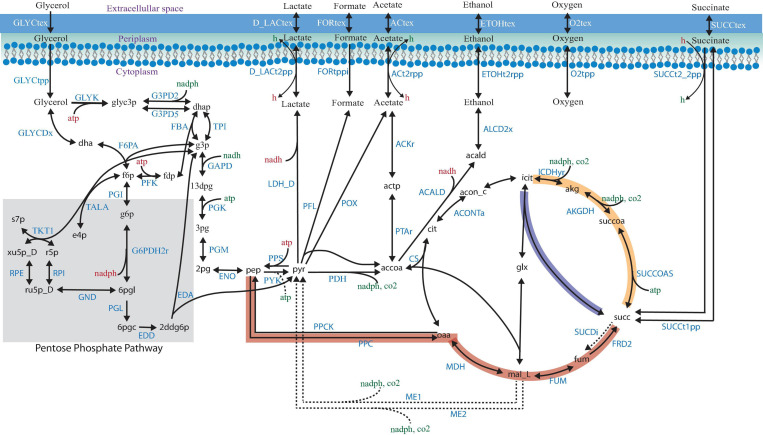
Succinic acid pathways from glycerol in *Escherichia coli*. The three pathways for succinic acid production are indicated by the thick red (the PEP–pyruvate–oxaloacetate node—the reductive TCA branch), yellow (the oxidative TCA branch), and blue (the glyoxylate shunt) arrows. Relevant biochemical reactions are represented based on the ID BIGG database names (King et al., 2016).

Shifting from petrochemical sources to renewable, abundant, and inexpensive feedstocks to obtain valuable chemicals has become a promising goal for the chemical industry (Vlysidis et al., 2011). The biodiesel industry has increased in the last years by using renewable raw materials, but it generates large amounts of glycerol, which has become a burden. The bioconversion of glycerol is a potential route to increasing the use of bio-based succinic acid, a critical building block chemical with an attractive market. The availability of three pathways for succinic acid production (Figure 1; Chen et al., 2013a), the adaptability to different environments, and the accessibility of metabolic engineering and omics tools make *Escherichia coli* an attractive cell factory. However, some challenges, such as low growth rate and yield, the use of a rich medium, the generation of by-products, and various anaerobic requirements, need to be overcome for bio-based succinic acid production, considering cost-effective issues, as compared with the petroleum-based approach.

The main goal of using microbial cell factories is to design cheap and high-yield biotechnology-based conversion processes. A significant problem to be solved is how to enhance cell growth while using its capabilities to obtain a high-yield target chemical product. A classical approach for that is adaptive laboratory evolution (ALE), which is based on the selection of microorganisms with superior production capability after random mutagenesis screening. Another approach to strain improvement is metabolic engineering, which uses genetic manipulation to optimize the production of desired compounds. Metabolic engineering selects targets that increase productivity based on the rationality of trial-and-error development cycles and an understanding of the routes playing a significant role in the synthesis. Strain design with this method has been extensively applied to use and/or produce interesting compounds (Kern A. et al., 2007; Chen et al., 2013a,b; Förster and Gescher, 2014; Woo and Park, 2014), including bio-based organic acids by substrate transport enhancement, gene overexpression, and deletion (Shams Yazdani and Gonzalez, 2008; Zhang B. et al., 2012; Buschke et al., 2013; Förster and Gescher, 2014; Yin et al., 2015; Zhu and Jackson, 2015). However, making the strain industrially competitive requires much time, effort, and high cost (Rangel et al., 2020).

When DNA was discovered in the last century, a new approach called metabolic network modeling for the study of cellular metabolism was developed (O’Brien et al., 2015). It allows to determine how several pathways in a cell can interact, as well as to elucidate basic microbial processes (Haggart et al., 2011). The first genome-scale metabolic network was described in 1999, and in 2002, the use of metabolic modeling to analyze recombinant pathways was reported (Carlson et al., 2002). Several models have been developed ever since with significant accuracy and useful predictions (Portela et al., 2013) that can be used to guide experimental studies (Pharkya and Maranas, 2006; O’Brien et al., 2015). COnstraints-Based Reconstruction and Analysis (COBRA) methods make it possible to predict, given a cellular objective function, attractive targets to increase or maximize biochemical yields, and to determine perturbations after genetic manipulations of the cell (Kim, 2012; Ruckerbauer et al., 2015). OptKnock, OptStrain, OptForce, and OptReg are some COBRA methods developed to predict metabolic engineering targets for cell optimization by using gene–protein reaction (GPR) relationship (Burgard et al., 2003; Pharkya et al., 2003; Pharkya et al., 2004; Pharkya and Maranas, 2006; Ranganathan et al., 2010).

OptKnock applies a flux balance analysis (FBA) approach for simulating genome-scale metabolic models (GEMs). It assumes that each organism’s metabolic network has been tuned through evolution for some objective function, be it a maximal growth rate or energy efficiency (e.g., minimal ATP utilization). While this assumption may be valid for wild-type (WT) organisms that have evolved over many hundreds or thousands of generations, it may be less appropriate for engineered mutants (KO) because they have been engineered in a controlled environment and unexposed to the same evolutionary forces. Hypothesizing that mutant organisms are unable to immediately adapt their metabolic network to achieve the WT objective function, computational tools such as minimization of metabolic adjustment (MOMA) were developed (Segre et al., 2002). This approach is mathematically formalized as a quadratic programming (QP) problem, finding a suboptimal flux profile that is a minimal Euclidean distance from the WT (WT-FBA) and the genetically perturbed (KO-FBA) organisms. FBA combined with MOMA evaluation after OptKnock prediction could provide a more accurate prediction of the immediate metabolic response to KO than FBA does on its own. However, a large list of knockout combinations could be obtained when computational tools are used, and select which test in a lab can be laborious.

Several approaches to optimize cell factories have been developed, but conventional and computational approaches have not always been successful due to unexpected changes in the cell where an intracellular complex interconnected network of genes, proteins, and reactions exists. Systems metabolic engineering has emerged as an approach that integrates metabolic engineering and combined metabolic and “omics” network models. This approach could be beneficial for genome-scale modeling because it reduces the solution space and generates accurate predictions (Nordlander et al., 2008; Feist et al., 2010; Blazier and Papin, 2012; Machado and Herrgård, 2014; Rangel et al., 2020). Mainly considering that under certain environmental conditions, there are a limited number of reactions that are active according to transcriptional responses and other regulation phenomena to provide beneficial improvements for the cell bioconversion process (Fong and Marciniak, 2003; Fong et al., 2005; Lee and Palsson, 2010; Conrad et al., 2011; Wang et al., 2011; Zhang J. et al., 2012; Bao et al., 2014).

In this study, systems metabolic engineering for overproduction of succinic acid from glycerol in *E. coli* ATCC 8739 was used through integration of transcriptomics data to metabolic models and classification tree analysis using the random forest to classify gene targets predicted by OptKnock. Our strategy took advantage of transcriptomics data obtained from an evolved *E. coli* in glycerol and an optimized culture media. These data were subsequently integrated into a metabolic network model to predict targets using OptKnock. Predicted combinations were then evaluated using FBA, flux variability analysis, and MOMA to determine the effects of gene reaction knockout in the cell. Finally, predicted target reactions were evaluated using random forest to determine the importance of each target using succinic acid production, growth rate, and Euclidian distance between the WT strain and each mutant as response variables.

## Materials and Methods

### Strains and Culture Conditions

*E. coli* ATCC 8739 was used in this study. It was obtained commercially from the American Type Culture Collection (ATCC). A glycerol-based medium containing the following components (per liter) was used as the reference culture condition: 5 g of yeast extract, 2.5 g of NaCl, 5 ml of trace metal solution [0.55 g/L CaCl_2_, 0.10 g/L MnCl_2_ 4H_2_O, 0.17 g/L ZnCl_2_, 0.043 g/L CuCl_2_ 2H_2_O, 0.06 g/L CoCl_2_ 6H_2_O, 0.06 g/L Na_2_MoO_4_ 2H_2_O, 0.06 g/L Fe(NH_4_)_2_(SO4)_2_ 6H_2_O, 0.20 g/L FeCl_3_ 6H_2_O], 5 ml of MgSO_4_ (1 M), and 30 g of glycerol. A 50 ml culture was carried out in a 250 ml baffled-conical Erlenmeyer flask and cultivated aerobically at 37°C and 200 rpm.

Two conditions were evaluated in this study: an adapted *E. coli* on high glycerol concentrations (30, 40, 50, 60 g/L) and an optimized culture condition. For the first condition, four *E. coli* cultures were continuously subcultured each for 72 h in Luria–Bertani (LB) medium (5 g/L of NaCl, 10 g/L of tryptone, and 5 g/L of yeast extract, supplemented with 30, 40, 50, or 60 g/L). After every three subcultured rounds (216 h), the concentration of tryptone was decreased from 1 until reaching 0 g/L. Then, 10 subcultured rounds each for 72 h were carried out. During the complete experiment, a 50 ml culture was carried out in a 250 ml non-baffled-conical Erlenmeyer flask and cultivated aerobically at 37°C and 200 rpm. For each subcultured round, an OD ∼0.33 600 nm was considered as inoculum starting point. At the end of each tryptone decreasing, 1 ml of culture was kept at –80°C and used for further evaluation of growth and glycerol uptake. For the optimized culture condition, the glycerol-based medium was supplemented with 1 g of NH_4_Cl, 6 g of Na_2_HPO_4_, and 3 g of K_2_HPO_4_ at the same conditions as the reference culture.

### Differential Expression Analysis

RNA-Seq was carried out in triplicate for all conditions. For the adapted strain, the culture conditions for RNA-Seq were the same as those for the optimized culture medium condition. To harvest cells for total RNA purification, the culture sample was first treated with RNAprotect Bacteria Reagent (Cat No./ID: 76506), and enzymatic lysis and proteinase K digestion of the bacteria were carried out following the manufacturer’s protocol. Then, the Qiagen RNeasy Mini kit (Cat No./ID: 74104), following the manufacturer’s protocol, was used to obtain the total RNA for further analysis. Each sample was treated with DNase following the protocol in order to remove the DNA. The samples were sent to commercial RNA-Seq services for further sample processing and sequencing (Genewiz, South Plainfield, NJ).

Clean, raw data was obtained by removing the reads containing adapters using Trimmomatic. The sequence RefSeq: NC_CP010468 was employed for mapping. RNA reads were mapped using the software bowtie2, and featureCounts was employed to read counts. SARTools (Statistical Analysis of RNA-Seq data Tools) (Varet et al., 2016) was used for statistical RNA-Seq analysis. Differentially expressed genes (DEGs) were identified using the DESeq2 R Package. The functional classification of the DEGs was performed using Gene Ontology (GO) analysis by Blast2GO (Götz et al., 2008). The data discussed in this publication have been deposited in NCBI’s Gene Expression Omnibus (Edgar, 2002) and are accessible through GEO Series accession number GSE140847.

### Genome-Scale Metabolic Network Reconstruction

In order to obtain metabolic engineering targets to overproduce succinic acid from glycerol, two *E. coli* models were used: *EColiCore2* (ECC2) (data under peer review) and iTA1338 for *E. coli* ATCC 8739 (Supplementary File 1). Gene associations for both models were modified to ECOLC_RS number based on the sequence RefSeq: NC_CP010468 to facilitate the integration of transcriptomics data. Extensive manual curation was conducted, including (i) adding/eliminating transport reactions and extracellular metabolites and (ii) filling pathway gaps. GapFind and GapFill, two optimization problems that search for root metabolite problems that are not connected in the network and that solve them, were used to fill gaps in iTA1338, including biomass reaction BIOMASS_Ec_iML1515_WT_75p37M (Supplementary File 1). All optimization problems were solved using the COBRA Toolbox v.3.0 (Heirendt et al., 2019).

### Transcriptomics Integration and Metabolic Engineering Target Prediction

The gene inactivity moderated by metabolism and expression (GIMME) (Becker and Palsson, 2008) method was used to integrate transcriptome data with the *E. coli* metabolic model. This method then minimized the usage of low-expression reactions while keeping the objective (e.g., biomass) above a certain value. Expressed genes were considered according to their expression level with log2 fold change (FC) ≥ |1|. Next, according to the GPR rules and the defined gene expression states, a specific activity state for each reaction was identified. Finally, a specific context model was obtained from the transcriptomic data. Metabolic engineering targets were obtained using OptKnock. However, MOMA was used to understand the probability of those mutants predicted to be adapted and to reach the optimal state (predicted succinic and growth flux) considering the Euclidean distance. It because OptKnock predicts an optimal state, but after genetic manipulation cell are not in this state. The maximum uptake rate of glycerol was set to 13.3 mmol/g DW h^–1^. The OptKnock, GIMME, and MOMA methods were conducted using COBRA Toolbox v.3.0 (Heirendt et al., 2019) in MATLAB 2017b and Gurobi 8.0.1.

### Machine Learning to Determine Potential Metabolic Engineering Targets

Random forest models are supervised machine learning approaches, which have the advantage of giving a summary of the importance of each variable. This approach is based on a randomized variable selection process. An estimation of variable importance is provided by *IncNodePurity*, which measures the decrease in tree node purity that results from all splits of a given variable over all trees (Li et al., 2015). For interpretation purposes, this measure can be used to rank variables by the strength of their relation to the response variable (Li et al., 2015). A matrix of binary values was built from *m* mutant predicted and *n* reactions in the set of possible reactions to be knocked out. In this matrix, one represents the presence of one specific reaction to be deleted in the mutant and zero the absence in the combination of reactions to be deleted in the mutant. The matrix was partitioned into training and test sets; the training set was used to build a random forest model to predict succinic acid production, growth rate, or the growth rate Euclidean distance between the mutant and WT strains as response variables. For the training set, succinic acid production, growth rate variable response was initially predicted using FBA, and the growth rate Euclidean distance between the mutant and WT strains was predicted using MOMA. Next, the model performance was assessed using the testing set. Finally, we used the random forest to determine the importance of each target reaction over the three evaluated response variables.

## Results

### Glycerol Consumption of *E. coli* After Adaptive Laboratory Evolution

Luria–Bertani is one of the most common cultures used industrially for the growth of *E. coli*. In order to increase glycerol consumption by *E. coli* on LB media, an ALE experiment was carried out. Results obtained in this study, before the ALE experiments, suggest that even when high cell density cultures are reached, a low consumption of glycerol is observed. For all the four conditions (supplementation of 30, 40, 50, or 60 g/L of glycerol), a growth curve was carried out, showing that a maximum of 7 g/L consumption of glycerol could be achieved naturally by *E. coli*. Nevertheless, after the ALE experiments, an increase of 3 g/L in the glycerol consumption was observed for the strain growing in a supplementation of 30 g/L of glycerol. Despite this data showing an increase of around 30% in glycerol consumption, it is far below that obtained in the optimized culture, which reaches a consumption of 30 g/L of glycerol (data under peer review).

### Transcriptional Response of *E. coli* for Aerobic Glycerol Consumption

A cell is considered a complex system where a large number of processes are carried out. These processes then involve an interaction between genes, transcripts, proteins, metabolites, and reactions, among others (Lee et al., 2012; Furusawa et al., 2013; Rangel et al., 2020). Metabolic models are reconstructed by using genome information; however, it is well known that metabolism is given by environmental conditions by passing through a cell regulation process. This causes some genes to be turned on and off under certain conditions. To determine which reactions are active to obtain high accurate models, two transcriptomic profiles were obtained from an ALE experiment and an optimized culture medium.

**FIGURE 2:**
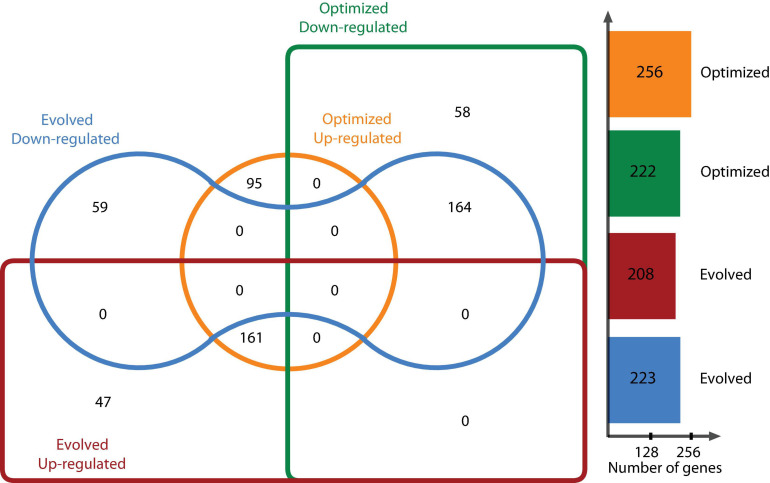
*E. coli* ATCC 8739 differentially expressed genes in the optimized medium vs. glycerol-based medium and the evolved strain vs. glycerol-based medium.

DEGs were determined using the DESeq2 statistical package after filtering out low count reads with an average value of <100. Significant DEGs were defined as those whose abundance had at least a log2 fold change [(log2 FC) > | 2|] between the reference condition (glycerol-based medium) and a chosen experimental condition (optimized culture medium and evolved strain) at a false discovery rate (FDR)-corrected *P* < 0.05. Relevant genes with log2 FC > | 1| for glycerol metabolism or under the same regulon were taken into account. Figure 2 shows the distribution of DEGs using a log2 FC ≥ |2| for one strain growing in the optimized culture medium and one evolved strain growing in the same optimized medium. This analysis determined that 478 genes were differentially expressed, with 222 genes downregulated and 256 upregulated for the optimized medium, and 431 DEGs for the evolved strain, of which 223 genes were downregulated and 208 genes were upregulated. When comparing DEGs in the optimized medium and those in the evolved strain, 59 downregulated genes were found to be unique in the evolved strain and 58 unique genes for the optimized medium. In this context, 47 and 95 upregulated genes were found to be unique in the evolved strain and the optimized medium, respectively (Figure 2).

**FIGURE 3:**
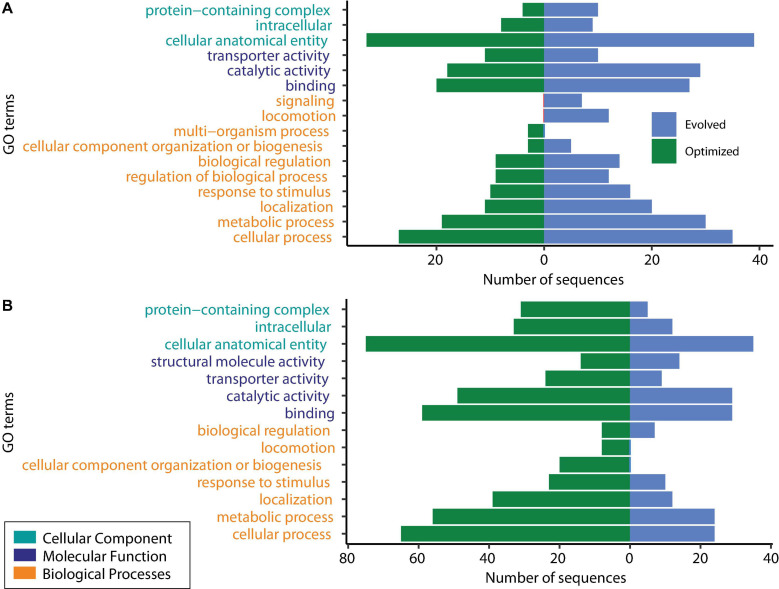
Gene Ontology (GO) analysis of unique differentially expressed genes. **(A)** Downregulated genes. **(B)** Upregulated genes.

DEGs were classified into the following three groups using GO analysis: biological processes, molecular functions, and cellular components. The shared downregulated genes predominantly included those involved in the metabolic process (cellular, organic substances, nitrogen compounds, and primary metabolic processes), chemicals, stress and stimulus responses, and heterocyclic compound systems. Between downregulated genes, we found *phoB* and *phoR*, which are involved in phosphorous uptake and metabolism since, under excess phosphorous, PhoR inactivates *phoB* (Makino et al., 1989). Figure 3 shows the level 2 GO terms for unique down- and upregulated genes in both conditions using Blast2GO (Götz et al., 2008). The 117 unique downregulated genes at log2 FC ≥ |2| and an adjusted *P* ≤ 0.05 were classified into 15 functional groups. Two GO terms, signaling and locomotion, were only present for the evolved strain, and one GO term, multiorganism processes, was only present for the optimized culture condition in downregulated genes (Figure 3A).

GO analysis revealed that shared upregulated DEGs (Figure 2) are involved mostly in the metabolic process (51%), including GO terms such as cellular, organic substances, primary, and nitrogen compound processes; 11% of the upregulated genes were associated with biosynthetic processes and the establishment of localization. The main GO terms for molecular functions were those involved in a binding activity (66%), counting ions, heterocyclic compounds, organic cyclic compounds, small molecules, and protein binding, followed by transferase activity (10%) and transmembrane transporter activity (9%). About 42% of the DEGs categorized in cellular functions were implicated in membrane GO terms, with 17% in the cell periphery and 16% in the cytoplasm.

Glycerol metabolism in *E. coli* is mediated by *glp* operons. In consequence, transcriptomic analysis shows shared upregulation of *glpBCFKQTX* genes. The changes in bacterial gene expression in response to glycerol utilization are summarized in Table 1. During glycerol utilization, GlpF permease facilitates glycerol entry into *E. coli* for further transformation into glycerol-3-phosphate (Gly-3-P) by GlpK under aerobic conditions. Comparing *glpK* expression with the values obtained for other genes in the glp regulon showed that *glpK* was one of the most highly expressed genes. However, a difference of ∼1 log2 FC between the evolved strain and the optimized culture condition was exhibited in the *glpFKX* operon (Table 1). As a consequence of the regulatory network, an increase in the expression of *glpX* was detected (2.76 log2 FC), which is part of the *glpFKX* operon and works as an alternative fructose-1,6-bisphosphatase involved in gluconeogenesis by catalyzing the hydrolysis of fructose-1,6-bisphosphate to fructose 6-phosphate (Booth, 2014). Overexpression of *glpX* has been shown to increase hydrogen production (Kim et al., 2011). Additionally, transcriptomic analysis showed upregulation of both flavin oxidases *glpD* and *glpABC*.

**TABLE 1:** Differential expression of genes involved in glycerol metabolism.

RefSeq tag (ECOLC_RS)	Gene name	Old locus tag	Product	Log2 FC Exp 3	Log2 FC evolved
01540	*glpD*	EcolC_0288	Aerobic glycerol-3-phosphate dehydrogenase	1.69	1.21
07540	*glpC*	EcolC_1408	Anaerobic glycerol-3-phosphate dehydrogenase subunit C	2.61	3.59
07545	*glpB*	EcolC_1409	Anaerobic glycerol-3-phosphate dehydrogenase subunit B	2.87	3.74
07550	*glpA*	EcolC_1410	Sn-glycerol-3-phosphate dehydrogenase subunit A	1.28	2.00
07555	*glpT*	EcolC_1411	Glycerol-3-phosphate transporter	5.41	4.36
07560	*glpQ*	EcolC_1412	Glycerophosphoryl diester phosphodiesterase	5.25	5.52
22045	*glpF*	EcolC_4091	Aquaporin	4.17	3.03
22050	*glpK*	EcolC_4092	Glycerol kinase	5.36	4.37
22055	*glpX*	EcolC_4093	Fructose-1,6-bisphosphatase	2.76	2.05
10840	*fumA*	EcolC_2018	Fumarate hydratase	1.46	−0.60
20740	*frdA*	EcolC_3856	Fumarate reductase flavoprotein subunit	0.68	1.71
20745	*frdB*	EcolC_3857	Fumarate reductase iron-sulfur subunit	0.89	1.87
20750	*frdC*	EcolC_3858	Fumarate reductase subunit C	0.74	1.72
20755	*frdD*	EcolC_3859	Fumarate reductase subunit D	0.16	1.09

The electron-transport chains of *E. coli* are composed of many different dehydrogenases and terminal reductases. Glycerol metabolism in *E. coli* uses oxygen as the main electron acceptor, but it could also employ fumarate under anaerobic conditions by encoding a fumarate reductase complex under anaerobic conditions (Jones and Gunsalus, 1987; Cecchini et al., 2002). Table 1 shows log2 FC for *fumA* and *frdABCD* genes in *E. coli*. The *fumA* gene was encoded for abundant fumarase, predominantly expressed in the optimized culture medium (1.55 log2 FC), but not for the evolved strain (−0.53 log2 FC). FumA has been reported to be predominantly expressed under aerobic conditions (Chen et al., 2012). Under aerobic conditions, the catalysis of succinate to fumarate interconversion is mediated by the succinate dehydrogenase complex encoded by *sdhABCD* (Cecchini et al., 2002). However, in this study, *sdhABCD* genes were not found to be differentially expressed in any of the culture conditions. Interestingly, among the upregulated genes in the adapted strain, a difference of ∼1 log2 FC in the expression of the fumarate reductase genes (*frdABCD*), which is used in anaerobic growth, was observed over the optimized culture condition.

The maltose operon of *E. coli* consists of genes that encode proteins involved in the uptake and metabolism of maltose and maltodextrins. These genes have been found to be highly associated with upregulation under glycerol utilization as a carbon source, and changes in the level of *glpK* transcription had a significant effect on *malT* transcription (Chagneau et al., 2001). In this study, *malEFKMTPQ* genes were shown to be upregulated in both conditions. For *malT*, the log2 FC was more highly expressed in the optimized culture condition than in the evolved strain. The same behavior was observed for *glpK*. Thus, a high expression of this regulon in this study could be presumably linked to the high expression of the *glpK* gene since they showed similar log2 FC.

As a result of glycerol metabolism, acetate is mainly generated. In our analysis, the phosphate acetyltransferase encoded by *pta*, which catalyzes the reversible conversion between acetyl-CoA and acetylphosphate (Lin et al., 2005; Blankschien et al., 2010), was found to be upregulated (∼2.30 log2 FC). Also, the *atpABCDEFGH* genes have a role in the generation of ATP from ADP and phosphate. These genes were observed to be upregulated, with similar log2 FC, except for *atpA*, which had a difference of around 1 log2 FC in the optimized culture medium with respect to the evolved strain.

### Predicting Potential Metabolic Engineering Targets for Succinic Acid Overproduction

Genome-scale metabolic models (GEMs) are defined as a complete set of reactions involved in cell metabolism, given by genome annotation, regardless of whether the annotated metabolic genes are expressed in a given environment. This assumption could be correct in genome-scale models because core models represent the central metabolism, but the full potential of GEMs remains unexploited mainly (Ataman et al., 2017). To avoid this situation and to evaluate the effects of using a core or a large model to predict metabolic engineering targets, three models were used: a core model (ECC2) and two models obtained after the integration of transcriptomics data that can help to elucidate the actual state of the metabolic network *in vivo* for further metabolic engineering.

### Metabolic Model Reconstruction and Transcriptomics Integration

For the integration process, a reconstruction of the metabolic model for *E. coli* ATCC 8739 was carried out based on the iEcolC 1368 (Monk et al., 2013), iEC1349_Crooks (Monk et al., 2016), and iML1515 models (Monk et al., 2017). Extensive manual curation was conducted to fill pathway gaps. Transport and exchange reactions were added or eliminated, enabling nutrient uptake and by-product secretions. Finally, the resulting model was designated iTA1338, and it involved 2,032 metabolites, 2,804 reactions, and 1,338 genes (Supplementary File 1). After that, using GIMME, context-specific metabolic networks were constructed departing from the iTA1338 model for two types of strains: (1) WT *E. coli* ATCC 8739 growing in an optimized culture medium (iTA818) (Supplementary File 1) and (2) *E. coli* ATCC 8739 strains evolved to grow on glycerol (iTA821) (Supplementary File 1). Manual curation was carried for the iTA821 model based on GapFind and GapFill results.

**FIGURE 4:**
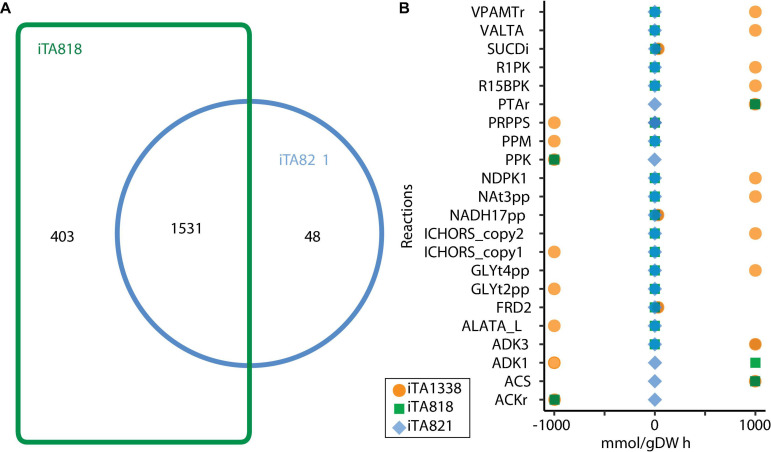
Models comparison after transcriptomic integration using gene inactivity moderated by metabolism and expression (GIMME) under aerobic conditions. **(A)** Venn diagram or reactions included in the model. **(B)** Flux Balance Analysis under aerobic conditions.

Figure 4 illustrates the number of reactions obtained for each model after transcriptomic integration. The same growth rate was observed after integration; however, flux distribution in 24 reactions was exhibited (Figure 4B). The reactions only present in iTA821 are mainly associated with the inner membrane transport (14). Other unique reactions in iTA821 were mapped to be linked to the citric acid cycle, cofactor and prosthetic group biosynthesis, glutamate metabolism, inorganic ion transport and metabolism, the nucleotide salvage pathway, oxidative phosphorylation, and pyruvate metabolism, among others. Unusual reactions of iTA818 were mainly associated with transport, including the transport outer membrane porin (218), transport inner membrane (50), and transport outer membrane (15), followed by cell envelope biosynthesis (37), the nucleotide salvage pathway (24), glycerophospholipid metabolism (14), alternate carbon metabolism (12), and cofactor and prosthetic group biosynthesis (7), among others.

### *In silico* Systems Metabolic Engineering Targets Prediction

To predict *E. coli* strains that overproduce succinic acid from glycerol, OptKnock was used (Burgard et al., 2003). Before predicting the reaction target to overproduce succinic acid, both metabolic networks were preprocessed. The goal of preprocessing was to obtain a smaller set of selected reactions that could serve as valid targets for gene knockouts. First, all reactions displaying maximum and minimum fluxes equal to zero were removed from the set of potential reactions to be knocked out. Next, all reactions that had been experimentally found to be essential for growth were removed from consideration (Joyce et al., 2006). Also, the reactions that were found to be computationally essential were not considered, as well as non-gene-associated reactions.

**FIGURE 5:**
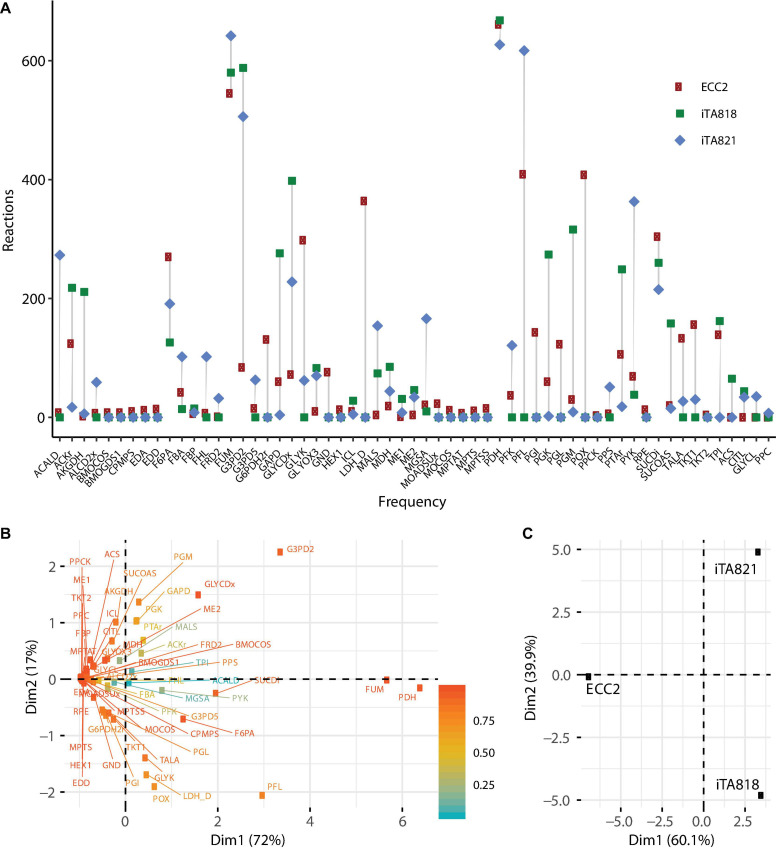
Metabolic engineering targets predicted by OptKnock. **(A)** Frequency of reactions predicted by OptKnock for each model, with combinations of knockouts from 1 to 10 reactions per mutant. **(B)** PCA for metabolic targets predicted. **(C)** PCA for models using predicted targets.

Ten OptKnock rounds of mutant prediction were carried out. In each round, the set of reactions was set up to 1, 2, 3, … 10, and 100 mutants were requested per round. ECC2, iTA818, and iTA821 models were used to predict mutants of succinic acid overproducers; 811, 806, and 785 possible mutants were obtained from the ECC2, iTA818, and iTA821 models, respectively (Supplementary File 2). Figure 5 describes the frequency of the reactions predicted in all the possible mutants. It can be seen that 30 reactions were above the average frequency. Reactions acetate kinase (ACKr), fructose 6-phosphate aldolase (F6PA), fumarase (FUM), pyruvate dehydrogenase (PDH), pyruvate formate lyase (PFL), phosphotransacetylase (PTAr), succinate dehydrogenase (SUCDi), triosephosphate isomerase (TPI), glycerol-3-phosphate dehydrogenase-NADP (G3PD2), and glycerol dehydrogenase (GLYCDx) were frequently predicted for the all models. It is important to mention that G6PDH2r, LDH_D, PGL, and POX were not predicted to be part of models iTA818 and iTA821 after integration.

Interestingly, in the complete set of reactions predicted, PDH was the most frequent target reaction, followed by FUM in all the models (Figure 5), and minimal variations in the knockout frequency were observed for these reactions. Figure 5A shows the plot of the first two principal components of the principal components analysis (PCA), representing the variability of 89% of the data. This analysis shows how PDH and FUM knockouts are closely related to succinic acid overproduction from glycerol. Regions of high variability are clustered along with the first principal component, presenting a value of zero for the first principal component. This indicates that the factors that make up the first principal component are critical for high titers. The contributions of different models to the first two principal components of the PCA are shown in Figure 5B, and they are indicative of the relative influence on the variability in knockout predictions given by transcriptomic integration.

**FIGURE 6:**
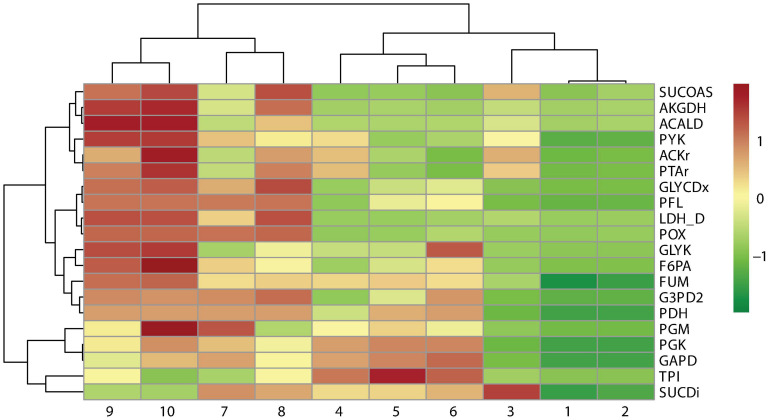
Pearson correlation of reaction frequency by knockout numbers (*k*), predicted using OptKnock.

A cluster analysis between the reaction frequency for each *k* deletion showed that elimination of acetate, formate, and lactate by-products mediated by POX, PFL, and LDH_D is highly related to PDH and FUM deletion (Figure 6). This phenomenon, probably due to PDH deletion, results in reduced conversion of pyruvate to acetyl-CoA, which is the main substrate in ACKr and PTAr reaction to generate acetate (Figure 1), a competitive by-product on succinate production (Blankschien et al., 2010). Then, if PDH deletion is not carried out, ACKr and PTAr knockouts would become essential to increasing succinate production, as well as minimizing costs in the separation process (Kurzrock and Weuster-Botz, 2010; López-Garzón and Straathof, 2014).

**FIGURE 7:**
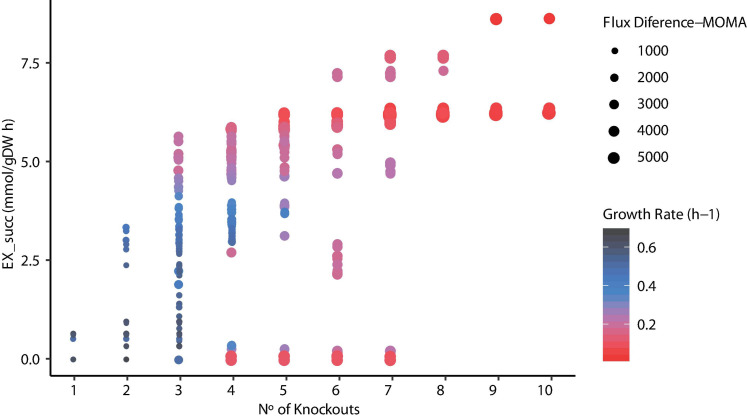
Relationship between the number of knockouts, succinic acid production, growth rate, and flux difference.

Since metabolic manipulation of cells results in a stressful process, the negative impact of deletions on the maximum growth rate can be observed. To determine the effects of reaction knockouts over the cell, FBA was carried out and Euclidean distance was calculated for each mutant predicted. Figure 7 illustrates the relationship between the number of knockouts, succinic acid production, and growth rate using FBA and the Euclidian distance between the WT and mutant strains using MOMA. It can be seen that the number of reactions knocked is highly related to high succinic acid production rates due to the elimination of competitive by-products, such as acetate, formate, and lactate, requiring at least three to four deletions. The highest succinate production (∼8.5 mmol/g DW h^–1^) was observed in mutants predicted in ECC2 when 9 or 10 reactions were deleted. However, this implies a substantial reduction in the growth rate to ∼4% compared with the WT strain. Thus, selecting these mutants is unrealistic for the industrial production of succinic acid. The same behavior in the reduction of the growth rate was observed for those mutants that required more than six deletions in mutants predicted in iTA818 and iTA821. In contrast, a considerable reduction in the growth rate (28% of the WT growth rate) as well as an increasing succinic acid production rate (around 30% more than those with 9–10 knockouts) for those mutants with six knockouts was observed. In addition, it was observed that there is no direct correlation, in the same magnitude for all the mutants, between the Euclidean distance and the numbers of knockouts in each mutant. However, Figure 7 shows amplifications in the Euclidian distance between the WT and the mutants when succinate production and knockout numbers increase and growth rate decreases.

### Identification of Critical Metabolic Targets and Potential Mutants

OptKnock results are a large list of knockout combinations where maximum product synthesis occurs at a maximum growth rate reachable (Burgard et al., 2003). However, it has been observed that the optimal solution of the target given by OptKnock is not necessarily growth-coupled, and some mutants predicted do not increase the product target. Consequently, selecting a mutant to be tested in the lab could be really difficult and probably result in a laborious process. Assuming that each mutant product growth “coupled” predicted will result in a successful biological production, these mutants can ensure high productivity over time and initially solve this situation (Shabestary and Hudson, 2016). To identify growth-coupled production solutions, a COBRA Toolbox function was used to verify the minimum and maximum production rates given a set of reactions to be knocked out. As a result, the same minimum and maximum flux for the desired product should be obtained when the maximum growth rate is achieved. One thousand seven hundred ninety-nine (1,799) mutants were predicted to be growth-coupled, 539 to be growth-coupled non-unique (maximum flux - minimum flux > 0.1), and 64 mutants were categorized as not growth-coupled (maximum flux < 0.1). For the mutants categorized as growth-coupled non-unique, an FBA was carried out to predict the succinic acid production rate (Figure 7), where 279 mutants were predicted to have a difference between the maximum production rate predicted by the function and FBA < 2, resulting in 2,078 *in silico* mutants that overproduce succinic acid.

**FIGURE 8:**
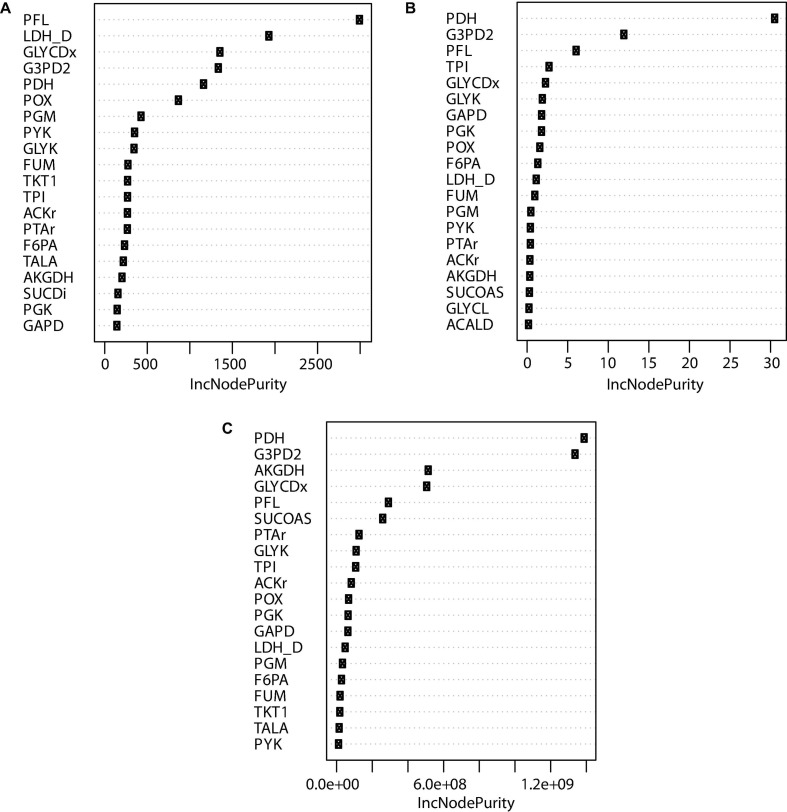
Top 20 node purities obtained using random forest for reactions predicted by OptKnock. **(A)** Reaction knockout importance on succinic acid production. **(B)** Reaction knockout importance in growth rate reduction. **(C)** Reaction knockout importance in Euclidian distance.

In order to filter and select potential mutants to be tested in the lab, a random forest model to predict the importance of each reaction knockout was developed based on the OptKnock predictions. Each possible combination of reactions using binary values that increase the succinic acid production was associated with the flux of the extracellular succinic acid and biomass reaction obtained by FBA and the Euclidian distance obtained by MOMA. The dataset was divided into two groups: 70% for training and 30% for the test. Following feature selection and cross-validation, a robust model that associated any combination of 58 reaction variables to a predicted growth rate and succinic acid production ratio was obtained. A measure of the importance of the contribution of each feature to the random forest model is shown in Figure 8 indicated by *IncNodePurity*. This model exhibited a mean square error (MSE) value of 0.293 when using the reaction flux of EX_succ_e flux obtained by FBA as a variable response. For growth rate (biomass reaction) as a response variable, the MSE value was 0.0002. Finally, when the Euclidian distance for each mutant was used as the response variable, the MSE value was 9,175.158, indicating that the Euclidian distance is not a good response variable to predict cell behavior when using the random forest model. Moreover, this result allows the use of machine learning models to predict the largest number of mutants than those obtained by OptKnock in terms of growth rate and succinic acid production since OptKnock is more time-consuming.

Figure 8A shows that PFL, LDH_D, GLYCDx, G3PD2, PDH, and POX are the most important reactions to increase the amount of succinic acid. These reactions are mainly associated with the GldA–DhaKLM fermentative route and the Gly-3-P route (Figure 1) in glycerol utilization (Blankschien et al., 2010), as well as acetyl-CoA generation given by the PDH knockout. In around 24% of the mutants predicted, a combination of GLYCDx and G3PD2 reactions was found to increase succinic acid production. However, POX and LDH_D reactions were not present in iTA818 and iTA821 models, and PDH, G3PD2, and PFL were also found to be the most important reactions, predicted to have an effect on growth rate (Figure 8B).

The pyruvate dehydrogenase complex is a critical connection point between glycolysis and the TCA cycle, both of which function during aerobic respiration through catalyzing the conversion of pyruvate to acetyl coenzyme A (acetyl-CoA) (Schutte et al., 2015). PDH deactivation results in PFL carrying the flux from pyruvate to acetyl-CoA (Khodayari et al., 2015). Simple reaction knockouts show that PDH deletion results in a growth rate reduction of ∼5%. Additionally, five reactions (FUM, GAPD, PGK, PGM, and TPI) were predicted to have the most significant reduction (8–10%) in growth rate during glycerol utilization. Of those reactions, only FUM has a significant positive effect over succinate production when this deletion was carried out alone. However, in mutants in which both FUM and PDH were predicted (59.45%), TPI appeared in around 12.60% (Figure 5). Then, the deletion of genes associated with TPI in addition to FUM and PDH reactions could negatively affect growth rate.

## Discussion

Glycerol metabolism in *E. coli* has been described in the literature (Murarka et al., 2008; Booth, 2014). However, cell changes are carried out as a response to stressful situations. In this study, two conditions were tested for transcription response in *E. coli* to further integrate to metabolic network modeling. Gene expression-wide analyses reveal how cells have the ability to avoid glycerol toxicity, increasing consumption. The most striking response to glycerol consumption and the possible mechanism to optimize succinic acid production from glycerol were revealed by the combination of the transcriptome, metabolic modeling, and machine learning analyses.

After glycerol incorporation in the cell mediated by GlpF, glycerol can be metabolized through two pathways. The first is mediated by the glycerol kinase GlpK through phosphorylation of glycerol to Gly-3-P, followed by GlpD activity under aerobic conditions, leading to dihydroxyacetone phosphate (DHAP) (Figure 1). The alternative pathway consists of an oxidation step by glycerol dehydrogenase (GldA) to yield dihydroxyacetone (DHA), followed by phosphorylation by DHA kinase (DhaK) to yield DHAP as well. In this study, overexpression of *glpK* was observed in both conditions, with a difference of around 20%. This result is not surprising since the GlpK-mediated reaction is a rate-limiting step in glycerol utilization (Herring et al., 2006). However, it has been observed that under the optimized culture conditions, the glycerol utilization rate is higher than that in the evolved conditions, suggesting that other mechanisms should exit in the cell to enhance glycerol utilization. Gly-3-P is the first intermediate between the glycerol pathway and the TCA cycle, as well as between the biosynthesis and catabolism of lipids; however, accumulation of Gly-3-P can become toxic. Thus, it is carefully regulated (Booth, 2014). The export of Gly-3-P could be mediated by *phoE* and *ompF* membrane porins; however, downregulation of *phoE* (−8.67 and −9.04 log2 FC for the optimized culture and the evolved strain, respectively) and upregulation of *ompF* (log2 FC 2.43) in the optimized culture suggest that it could play an essential role in *E. coli* ATCC 8739 glycerol metabolism at high uptake rates avoiding toxicity.

The marked upregulation of *glpQ* (5.351 and 5.597 log2 FC for the optimized culture and evolved strain, respectively), which catalyzes the hydrolysis of glycerol-phosphodiesters to alcohol plus Gly-3-P together with *ompF*, could explain the partially higher transcript abundance of *glpT* since the externally generated (or supplied) Gly-3-P activates GlpT (Wong and Kwan, 1992; Lemieux et al., 2005). This protein exchanges Gly-3-P for phosphate, avoiding the toxicity of both Gly-3-P and the inorganic phosphates (Booth, 2014). As a result and considering that phosphate is necessary to increase glycerol utilization, autoregulation of the PhoB/PhoR two-component regulatory system needs to be down-expressed. Downregulation of PhoB/PhoR was observed in this study, which could explain the achievement of optimal density (Gao and Stock, 2013), as well as contribute to the regulation of glycerol phosphate metabolism (Baek and Lee, 2007).

The transcriptional analysis also identified the differential expression of both flavin oxidases *glpD* and *glpABC*. Once Gly-3-P is in the cytoplasm, it is oxidized to dihydroxyacetone phosphate by one of two flavin-dependent oxidases encoded by *glpD* or *glpABC* genes under aerobic or anaerobic conditions, respectively (Blankschien et al., 2010; Booth, 2014). In the presence of oxygen or nitrates, GlpD transfers electrons to the respective terminal oxidized. In contrast, under anaerobic conditions, the GlpABC system transfers the electrons to fumarate or nitrates (Unden and Bongaerts, 1997). GlpD upregulation was expected since culture conditions were under aerobic conditions, but a higher expression of the *glpABC* system was surprising. Overexpression of *glpABC* under aerobic conditions could be elucidated because of the activation of fumarate reductase enzymes (Table 1) in the evolved strain as a result of high cell densities during the ALE process. However, in glycerol fermentation studies, the Δ*frdA* mutant has been shown to be beneficial for glycerol fermentation because it prevents the negative impact of hydrogen by maintaining suitable redox conditions (Murarka et al., 2008). Moreover, its activity could be supported by *sdhABCD* since they are structurally and functionally homologous (Guest, 1981). Therefore, we hypothesized that *frdABCD* upregulation could be the reason why enhancement in glycerol utilization was not observed in the evolved strain, even when an optimized culture medium was employed.

Insights on the molecular adaptive responses of *E. coli* to glycerol consumption revealed by the transcriptional datasets identified a marked *hdeAB* upregulation only in the evolved strain. This is attractive since HdeAB are periplasmic proteins that play a role in optimal protection at low pH (Masuda and Church, 2003; Kern R. et al., 2007). Therefore, differences in *hdeAB* upregulation in the evolved strain and the optimized culture medium probably occur because acetate is the main product in glycerol utilization, and under ALE conditions, pH was not controlled. Moreover, the addition of a phosphate buffer system using the salts Na_2_HPO_4_ and KH_2_PO_4_ provides the culture medium used directly for the optimized condition with a buffering capacity.

It was observed that the main and preferable route for glycerol consumption is the pathway mediated by GlpK since this gene was highly overexpressed in high glycerol consumption cultures. Moreover, *glpK* deletion has also been observed to be essential for glycerol utilization as the sole carbon source (Velur Selvamani et al., 2014). Then, the deletion of this gene could result in a non-effective bioconversion process. As a result, this gene should not be taken into account for engineered *E. coli* strains using glycerol as the carbon source even when the GLYK reaction was repeatedly predicted to be knocked by OptKnock in ECC2 and iTA821 since two pathways for glycerol utilization in *E. coli* exist.

Based on OptKnock and random forest model predictions, four critical control points, glycolysis, pyruvate metabolism, the pentose phosphate pathway, and the TCA cycle, are associated with the overproduction of succinic acid. FUM and SUCDi appear to be the most significant keys in the TCA cycle for succinate overexpression. The results of this study suggest that they are mutually exclusive. Parallelly, the knockout of by-products such as acetate, formate, and lactate by deleting POX, ACKr, PTAr, PFL, and LDH_D was highly predicted to be knocked out. Those results are interesting since one of the bottlenecks for industrial production of bio-based products is the elimination of by-products, which could facilitate the recovery and purification process. These results and those obtained in the transcriptional responses suggest that deletion of the *pta* needs to be, almost as mandatory, carried out since acetate production becomes a competitive pathway in glycerol metabolism for succinic acid production (Zhang et al., 2010).

The pyruvate dehydrogenase complex is a critical connection point between glycolysis and the TCA cycle, both of which function during aerobic respiration through catalyzing the conversion of pyruvate to acetyl coenzyme A (acetyl-CoA) (Schutte et al., 2015). PDH deactivation results in PFL carrying the flux from pyruvate to acetyl-CoA (Khodayari et al., 2015). Simple reaction knockouts show that PDH deletion results in a growth rate reduction of ∼5%. Additionally, five reactions (FUM, GAPD, PGK, PGM, and TPI) were predicted to have the most significant reduction (8–10%) in growth rate during glycerol utilization. Of those reactions, only FUM has a significant positive effect over succinate production when this deletion was carried out alone. These results indicate that those mutants predicted by OptKnock, where FUM and PDH are predicted, need to betested in the lab because it has been observed that a low growth rate could negatively affect the profitability of industrial bio-based production products (Chen et al., 2013a; Tafur Rangel et al., 2018). However, in mutants in which both FUM and PDH were predicted (59.45%), TPI appeared in around 12.60% (Figure 5). Then, the deletion of genes associated with TPI in addition to FUM and PDH reactions could negatively affect the growth rate. This is because in the absence of TpiA, DHAP is converted to methylglyoxal, which, even at submillimolar concentrations, is a toxic compound (Booth, 2014). DHAP is the result of the alternative pathway on glycerol metabolization consisting of an oxidation step by glycerol dehydrogenase (GldA). DHAP must be transformed into the general glycolytic pathway through isomerization by triosephosphate isomerase (TpiA) as glyceraldehyde-3-phosphate (GA3P). Therefore, deletion of *tpiA* could result in growth inhibition and cell death in the presence of glycerol as the only carbon source (Velur Selvamani et al., 2014). However, since FBA is not able to capture regulation, this situation could not be predicted by OptKnock.

Finally, computational models suggest that deletions of just six to seven reaction knockouts are beneficial for industrial production since the growth rate does not decrease extremely. It is important to consider that a similar succinate production could be achieved if six to eight reactions are knocked out for all models. An assumption using optimization methods to predict cell capabilities is that the cell could quickly adjust the metabolism to maximize growth under certain conditions. This affirmation could be true for WT strains because FBA predicts an optimal condition. However, in metabolically engineered strains, the cell attempts to compensate for the genetic changes carried out by the fewest changes in gene regulation until it achieves an optimal state that could be predicted using FBA (Senger et al., 2015). Then, FBA in engineered strains predicts a long-term evolved state. Thus, an alternative to evaluate unevolved mutants is the MOMA method (Segre et al., 2002). MOMA solves this problem by finding the solution that is most similar to the WT state (maximization of WT growth rate). Figure 7 shows a jump in the Euclidian distance between the WT and mutant strains when succinate production increases. This result could imply that after genetic manipulation, microbial cell factories require to be evolutionarily engineered. ALE studies have shown to provide the cell with the ability to grow under selection pressure to go up from a suboptimal state to optimal growth rate predicted using *in silico* models (Ibarra et al., 2002). Moreover, since OptKnock seeks to maximize the flux of a target chemical while maximizing the growth rate, our predictions could be beneficial for further ALE experiments because microbial cell factories have naturally evolved to maximize the growth rate. Thus, the succinic acid production rate would increase as biomass formation increases (Shabestary and Hudson, 2016) by using ALE rounds after metabolically engineering cells (Graf et al., 2019).

## Conclusion

By adopting tools from various disciplines, computational methods for systems metabolic engineering have been developed to understand cell behavior and how level systems (RNA, proteins, and metabolites, among others) can interact inside the cell for industrial purposes. In the same way, *E. coli* has been extensively studied to become a cell factory for the production of useful bio-based chemicals and materials through its native capabilities. However, there are some challenges that still need to be overcome.

This study proposes that computational tools can accelerate the optimization of cell factories by identifying metabolic engineering targets (genes/reactions) and not just by predicting mutants that may be biologically unviable. Therefore, systems metabolic engineering reduces time in rational strain design and guides in the selection of metabolic engineering targets based on cell behavior under experimental conditions. Simultaneously, departing from traditional computational tools, new methods such as machine learning could be proposed as an interesting alternative for the reduction of computational demand. However, these techniques are dependent on the level of completeness and accuracy of the metabolic model considered, which could be improved by using omics data.

## Data Availability Statement

The original contributions presented in the study are included in the article/Supplementary Material. The RNA datasets presented in this study can be found in the NCBI’s Gene Expression Omnibus database and are accessible through GEO Series accession number GSE140847. Further inquiries can be directed to the corresponding author/s.

## Author Contributions

AT: conceptualization, methodology, software, validation, investigation, visualization, formal analysis, and writing—original draft preparation. WR, DM, and CO: investigation. RC: methodology, software, and writing—review and editing. JG: supervision and writing—review and editing. AG: conceptualization, supervision, and writing—review and editing. All authors contributed to the article and approved the submitted version.

## Conflict of Interest

The authors declare that the research was conducted in the absence of any commercial or financial relationships that could be construed as a potential conflict of interest.
